# Disparities in the utilisation of preventive health services by the employment status: An analysis of 2007-2012 South Korean national survey

**DOI:** 10.1371/journal.pone.0207737

**Published:** 2018-12-26

**Authors:** SangJune Kim, Jee Hey Song, Yoo Min Oh, Sang Min Park

**Affiliations:** 1 London School of Economics and Political Sciences, London, United Kingdom; 2 Seoul National University College of Medicine, Seoul, South Korea; 3 Department of Family Medicine & Department of Biomedical Sciences, Seoul National University College of Medicine, Seoul, South Korea; Makerere University School of Public Health, UGANDA

## Abstract

**Objectives:**

This study aims to investigate the differences in the utilisation of preventive health services among standard, nonstandard workers, the self-employed, and unpaid family workers.

**Methods:**

We used the 4^th^ and 5^th^ Korea National Health and Nutrition Examination Survey, a nationwide survey conducted from the year 2007 to 2012. Economically active workers between the ages of 25 and 64 were grouped into standard, nonstandard, the self-employed, and the unpaid family workers (N = 16,964). Outcome variables are the uptake of preventive health services including influenza vaccination, regular medical check-up, and four types of cancer screenings. We used multivariate logistic models.

**Results:**

Overall, non-standard workers, the self-employed, and unpaid family workers were less likely to use the preventive health care compared to the standard workers. In particular, the self-employed were less likely to use all the six services compared to the standard workers and showed the lowest level of uptakes among the four working groups. Moreover, the service uptake of the non-standard workers was lower than that of standard workers in all services; except the colon cancer screening. On the other hand, unpaid family workers showed mixed results. While the uptake of influenza vaccination and regular health screening were lower, participation to the cancer screening was not lower compared to that of standard workers.

**Conclusion:**

There were gaps in the utilisation of preventive services among workers depending on their employment types. Access to preventive health care services of nonstandard workers, the self-employed, and unpaid family workers should be prioritised.

## Introduction

Labour conditions are essential social determinants to health [1–4]. It is a well-known fact that unemployment and the change of employment status matter not only to the mental health of the workers [5–7] but also to the conditions such as; cardiovascular disease [8] and even mortality [9]. Even in the cases of employment, studies have shown that precarious employment is associated with poor self-rated health [1, 10, 11] and mental health [1, 10, 12], compared to full-time permanent work.

From the governmental perspective, improving the workers’ health and reducing the health gap among the workers are two essential issues [4, 13]. According to the World Health Organization (WHO), the policies to protect workers from precarious environments target both health risks at the workplace and access to the health service [14]. Governments could mandate the health and safety regulations, the better management of hazardous materials, a smoking ban, and healthy workplace initiative. Moreover, governments could provide good access to preventive health services for workers in various ways [14]. Nonetheless, a pitfall in this approach is that not many workers are working in the accountable workplaces where the government can intervene. For example, more than 85% of workers in the world are working in small firms, informal sectors, and agriculture [15]. In fact, more than 80% of workers do not have access to the essential occupational health services worldwide [16].

Health gaps among workers also do matter. Literature shows that ‘non-standard workers’, including part-time, temporary, and daily workers, are more likely to have poor self-rated health [17] and poor mental health [18]. Regarding health service utilisation, these workers fall behind in both access to the regular clinic visits [19] and preventive health services such as cancer screenings [19, 20].

In South Korea, the estimated coverage of occupational health services (OHS) was about 70% in the early 2010s [21]. The labour law in South Korea states that every company should provide workers medical health screenings every six months, every year, or every two years depending on the level of risk of the working environment. Nonetheless, there are still many jobs without a full-time permanent contract in South Korea. The proportion of temporary workers, who work on contracts that span less than a year, was 24.7% in 2014, which is much higher than the average in OECD countries, 12.2% [22]. Moreover, the majority of the self-employed workers run small businesses in South Korea [23]. And 5% of the total population work in the agriculture industry [24]. Theoretically, these workers can get comprehensive preventive services through social health insurance named “National Health Insurance Service (NHIS)”. However, this is entirely voluntary.

Despite the comprehensiveness of previous studies on the preventive health utilisation of workers, the self-employed and unpaid family workers have not been accounted for [19]. A study using only the sample of the self-employed suggests that the self-employed have reasonably good self-rated health, utilize health care well, and get regular health checks compared to the general population. However, it does not hold the representativeness of the whole population [23].

The study investigates the gaps in the preventive health care use by the employment types. Section 2 sketches a general background regarding the preventive services. Section 3 describes the methodology. Section 4 provides the results of the empirical analysis. Finally, Section 5 discusses the implications of the findings and the limitations of the study.

## Background

### Preventive health services in South Korea

South Korea has achieved universal population health coverage in 2000 with the National Health Insurance Service (NHIS). In addition to the role as social health insurance, the NHIS has promoted the preventive health further to include three main programs; regular health check-ups, cancer screenings, and vaccine programs.

First of all, people receive medical check-ups in three different ways; NHIS-type for employees, NHIS-type for non-employees, or the private voluntary type. As stated above, all employees are supposed to receive the ‘NHIS-type’ medical check-up. It provides basic health check-ups that include; general consultation, blood pressure, lab test, eye and oral examination, and chest x-ray. The interval of check-up depends on the type of work and the risk of the work environment. Workers working in dangerous environments need to get evaluated every six months while others are checked annually [25]. The overall participation rate of workers in this type of medical check-up was 56% in 2006 and reached 72% in 2013 [26]. The other type is the ‘NHIS-type for local subscriber’. People in this group receive an invitation letter from the NHIS to receive a medical check-up, which is not mandatory. Some people opt for ‘voluntary medical check-up’ by paying the full cost of exams out of their own pockets.

Secondly, the NHIS has been providing cancer screenings since 1999. It is not explicitly designed only for the working population, but for the entire population. Since the year 2005, anyone who wa-s eligible for each cancer screening could receive a screening, either free of charge for medical aid recipients or with a 10% user-charge. The trend of cancer screening rates for all four cancers (stomach, colon, breast, and cervix) had increased steadily between 2004 and 2011 [27]. Data from 2011 show that the percentages of getting stomach cancer screenings and colon cancer screenings were 65.6% and 33.3%, respectively [27]. Every other year, women over 30 years old can be screened for cervical cancer with a Pap smear, and women over 40 years old are eligible for a mammography [28]. In 2011, the percentage of women who had the mammogram and Pap smear were 60.4% and 62.4%, respectively [27].

Lastly, the National Immunization Program (NIP) provides essential vaccination for children and seasonal influenza vaccination for high-risk groups. For the influenza vaccination, those over 65, the low-income, the disabled, and soldiers are eligible for free vaccinations while the others need to pay about 15–25 USD (1 USD: 1000 KRW) [29]. The vaccination rate during the 2008–2009 flu season was 32.4% for male and 42.6% for female. As intended, over 75% of the people over 65 years old received the vaccination. Vaccination rates were higher in rural areas (47.1%) compared to those in urban (31.5%) or metropolitan (30.7%) areas since the residential locations of the elderly population eligible for free vaccination are more concentrated in rural areas [29].

## Methodology

### Data

The Korea National Health and Nutrition Examination Survey (KNHANES) is a nationally representative cross-sectional health survey conducted by the Korea Center for Disease and Control and Prevention in South Korea. Beginning in 1998, KCDC had carried out the survey once every three years, then made it annual in 2006. It used a multi-stage clustered probability design using 192 primary sampling units (PSUs) from over 200,000 PSUs. The survey comprises four different sections including a health survey, health behaviour survey, lab data, and nutrition survey. All respondents have agreed to the use of provided information and contact for a potential follow-up. Detailed information about the survey is available [30]. The data is available from the website (https://knhanes.cdc.go.kr). Since the data, which is publicly available, were analysed anonymously, the institutional review board approval was waived for this study.

### Definition of the groups and study population

We categorised workers into four groups. ‘Standard workers (SWs)’ refers to workers with a full-time and permanent contract. ‘Nonstandard workers (NSWs)’ include part-time jobholders, temporary, and daily workers. Those who run their businesses are defined as ‘the self-employed (SE)’ [31]. Lastly, ‘unpaid family workers (UFWs)’ are also included.

The study periods were between 2007 and 2012. We used the 4^th^ and 5^th^ of KNHANES. The total number of the participants were 23,632 and 24,173, respectively. Due to the limited age between 25 and 64, the total population was 27,498. Among these, only economically active workers were included in the study population. (N = 16,964)

### Variables

Table 1 describes the definitions of the variables. We adopted six binary outcome variables to measure the use of preventive healthcare service that is in line with existing national guidelines. These include the regular medical check-up [25], stomach cancer screening, colon cancer screening, cervical cancer screening [32], and breast cancer screening within the past two years [33], as well as the influenza vaccination in the previous year [34]. Among the 16,964 respondents for each question, three answered “unsure” whether they had the influenza vaccine or not within the given period. For the regular health screening, only two people marked as “unsure”. Regarding the cancer screenings, 42 people for the stomach cancer screening and 78 people for the colon cancer have answered “unsure,” which is 0.24 per cent and 0.46 per cent of the total respondents. These respondents were excluded in each logistic regression.

**Table 1:** Variables used in the analysis.

Categories	Variables	Description
***Outcome***	**Health screening**	1: participated regular health screenings within two years 0: otherwise
	**Vaccination**	1: if vaccinated within one year 0: otherwise
	**Stomach cancer screening**	1: if participated stomach cancer screenings within two years 0: otherwise
	**Colon cancer screening**	1: if participated colon cancer screenings within two years 0: otherwise
	**Cervical cancer screening**	1: if participated cervical cancer screenings within two years 0: otherwise
	**Breast cancer screening**	1: if participated breast cancer screenings within two years 0: otherwise
***Types of employment***	**Type**	0: if full-time permanent job (SW) 1: if part-time, temporary, or daily employment (NSW) 2: if self-employed (SE) 3: if an unpaid family worker(UFW)
***Predisposing factors***	**Age**	
	**Female**	1: if female 0: otherwise
	**Education**	0: if primary school 1: if secondary school 2: if higher education
	**Marital status**	0: if married and stayed with the spouse 1: if never married 2: otherwise
	**Medical aid**	1: if medial aid recipient 0: otherwise (National Health Insurance)
***Enabling factors***	**Income**	The quartile of income ranged 0 (poorest) to 3 (richest)
	**Suburban**	1: if living in suburban area 0: otherwise
	**Private health insurance**	1: if had at least one indemnity private health insurance; 0: otherwise
***Need factors***	**Chronic disease**	1: if had at least one chronic disease such as hypertension, stroke, hypertension, stroke, myocardial infarction, angina, diabetes mellitus, chronic kidney disease, hepatitis B, hepatitis C, and liver cirrhosis 0: otherwise
	**Depressed mood**	1: if had depressive feeling more than two weeks 0: otherwise
***Work-related factors***	**Manual work**	1 if had manual work; 0 otherwise
	**Shift**	0: if had fixed working hours during the day 1: if had fixed working hours during the evening or night 2: if pulled shifts or had split working hours
***Year***	**2007 to 2012**	Dummy variables for each year
***Eligibility criteria for the NHIS cancer screening***	**Over 40**	1: if age > = 40 0: otherwise (for stomach cancer and breast cancer)
	**Over 50**	1: if age > = 50 0: otherwise (for colon cancer only)

We used the Andersen Model which explains the gaps in healthcare access [35]. The original model includes predisposing, enabling, and need characteristics. Since this study focuses on the preventive healthcare service utilisation of workers, we added work-related factors and eligibility criteria for the screenings.

### Statistical analysis

Since the outcomes are binary, all the models are multivariate logit models. We used STATA/SE 14 (StataCorp, College Station, TX, USA).

## Results

Table 2 presents descriptive statistics on various factors (i.e. Gender, socioeconomic status, health conditions, work conditions etc.) by the employment group.

**Table 2:** The number of observations and percentage proportion of the factors by each employment type.

Variables	Standard workers		Non-standard workers		Self-employed		Unpaid family workers		Total		
	N	Row %	N	Row %	N	Row %	N	Row %	N	Col %	Chi^2^
***Gender***											p<0.01
Male	4535	49.33	1061	11.54	3403	37.02	194	2.11	9193	54.95	
Female	2696	35.77	1981	26.28	1848	24.52	1013	13.44	7538	45.05	
***Quantiles of income***											p<0.01
1 (Poorest)	1457	29.78	1372	28.05	1611	32.93	452	9.24	4892	29.56	
2	1588	44.77	624	17.59	1100	31.01	235	6.63	3547	21.43	
3	2580	51.73	687	13.78	1432	28.71	288	5.78	4987	30.14	
4 (Richest)	1533	49.10	324	10.38	1043	33.41	222	7.11	3122	18.87	
***Marital status***											p<0.01
Married and stayed together	5547	41.24	2234	16.61	4532	33.70	1137	8.45	13450	80.53	
Others	393	32.13	413	33.77	394	32.22	23	1.88	1223	7.32	
Never married	1279	63.04	384	18.93	321	15.82	45	2.22	2029	12.15	
***Area of residence***											p<0.01
Urban	6173	47.75	2542	19.66	3629	28.07	585	4.52	12929	77.28	
Suburban	1058	27.83	500	13.15	1622	42.66	622	16.36	3802	22.72	
***Education***											p<0.01
Primary	391	16.02	649	26.59	942	38.59	459	18.80	2441	14.59	
Secondary	2977	36.87	1709	21.17	2773	34.34	615	7.62	8074	48.26	
Higher education	3863	62.17	683	10.99	1535	24.70	133	2.14	6214	37.15	
***Medical-aid Beneficiaries***											p<0.01
No	7160	43.56	2884	17.55	5197	31.62	1196	7.28	16437	98.72	
Yes	43	20.19	123	57.75	38	17.84	9	4.23	213	1.28	
***Indemnity Private Health Insurance (PHI)***											p<0.01
Without PHI	750	29.93	618	24.66	874	34.88	264	10.53	2506	15.22	
With PHI	6360	45.56	2370	16.98	4298	30.79	932	6.68	13960	84.78	
***Diagnosed with at least one chronic disease***											p<0.01
No	5847	45.83	2316	18.15	3754	29.42	841	6.59	12758	76.25	
Yes	1384	34.84	726	18.27	1497	37.68	366	9.21	3973	23.75	
***Depressive symptom lasting more than two weeks***											p<0.01
No	6584	44.76	2526	17.17	4588	31.19	1013	6.89	14711	87.98	
Yes	642	31.96	511	25.44	662	32.95	194	9.66	2009	12.02	
***Manual work***											p<0.01
Non-manual	4070	65.69	702	11.33	1300	20.98	124	2.00	6196	37.11	
Manual	3140	29.90	2334	22.23	3945	37.57	1081	10.30	10500	62.89	
***Work shift***											p<0.01
Day	6061	44.04	2399	17.43	4244	30.84	1057	7.68	13761	85.72	
Evening or night	273	20.15	318	23.47	671	49.52	93	6.86	1355	8.44	
Shift work or Split work	658	70.15	152	16.20	109	11.62	19	2.03	938	5.84	
***Augmented to other company***											p<0.01
No	6870	72.49	2607	27.51							
Yes	349	45.92	411	54.08							

Most study participants were married (80.53%), were living in the urban area (77.28%), had the National Health Insurance services (98.72%), had private health insurance (84.78%), were never diagnosed with a chronic disease (76.25%), were free from notable depressive symptoms (87.98%), and had day duties (85.72%).

Each employment group showed several distinguishable proportions for the given factors. The proportion of SWs was prominently higher in the “urban” group (47.75%), “higher education” group (62.17%), and “non-manual” working condition group (65.69%). SWs were also the majority among the population “with National Health Insurance” group (43.56%), as well as those “with private health insurance” group (45.56%). Only a small proportion of the participants were the recipients of Medical-aid (1.28%), but interestingly 57.75% of the recipients were NSWs. The groups that the SE took comparatively higher proportion were the “suburban” group (42.66%), the “primary education” group (38.59%), and the “evening or night duty” group (49.52%). The groups that UFWs had relatively greater percentage were the female group (13.44%), the “suburban” group (16.36%), and the “primary education” group (18.80%). Regarding income, the highest percentile of the “richest” group were SWs (49.10%), while there was not much difference in the “poorest” group for the proportions of SWs, NSWs, and the SE (29.78%, 28.05%, 32.93%).

Table 3 presents the proportion of receiving preventive services by the employment status.

**Table 3:** Utilization proportions of preventive health services by employment type.

Outcomes		Standard workers	Non-standard workers	The self-employed	Unpaid family workers	Total
**Regular health check**	**No**	0.2737	0.4792	0.5109	0.4662	0.3945
	**Yes**	0.7263	0.5208	0.4891	0.5338	0.6055
**Vaccination**	**No**	0.7562	0.7813	0.7898	0.7402	0.7701
	**Yes**	0.2438	0.2187	0.2102	0.2598	0.2299
**Stomach cancer screening**	**No**	0.6637	0.6687	0.6203	0.5506	0.6447
	**Yes**	0.3363	0.3313	0.3797	0.4494	0.3553
**Colon cancer screening**	**No**	0.8434	0.8309	0.7957	0.7679	0.8222
	**Yes**	0.1566	0.1691	0.2043	0.2321	0.1778
**Cervical cancer screening**	**No**	0.5277	0.5159	0.4952	0.4648	0.5095
	**Yes**	0.4723	0.4841	0.5048	0.5352	0.4905
**Breast cancer screening**	**No**	0.6375	0.5822	0.554	0.4865	0.5851
	**Yes**	0.3625	0.4178	0.446	0.5135	0.4149

Regarding regular health checks, about 72.63% of SWs received the medical check-up while the other three groups were marked between 48.91% to 53.38%. With the vaccination, all four groups ranged between 21.02% and 25.98%. Regarding cancer screening, UFWs seemed to have the highest proportion of getting screened, followed by the SE. SWs and NSWs showed lower proportions of getting cancer screening in all four areas.

Table 4 shows the gaps in the use of preventive services by the employment status. In fully-adjusted models, the probability of getting the influenza vaccination was 26% lower in NSWs (aOR = 0.74; 95%CI, 0.64–0.84), 29% lower in the SE (aOR = 0.7; 95%CI, 0.63–0.80), and 31% lower in the UFWs (aOR = 0.69; 95%CI, 0.57–0.83) than those of SWs. Moreover, the gaps between SWs and the others in the regular health screening were prominent. In particular, the SE (aOR = 0.25; 95%CI, 0.23–0.28) showed the lowest uptake, which was followed by UFWs (aOR = 0.32; 95%CI, 0.27–0.38) and NSWs (aOR = 0.40; 95%CI, 0.35–0.46). Moreover, NSWs (aOR = 0.81, 95%CI, 0.71–0.93) and the SE (aOR = 0.72, 95%CI 0.65–0.81) were less likely to get stomach cancer screenings compared to SWs. For the colon cancer screening, only the SE showed the lower uptake (aOR = 0.81; 95%CI, 0.71–0.93).

**Table 4:** Logistic regression analyses of four preventive health services for both male and female workers.

Types of preventive health service	Regular health screening within two years		Influenza vaccination within a year		Stomach cancer screening within two years		Colon cancer screening within two years	
Variables	aOR†	aOR††	aOR†	aOR††	aOR†	aOR††	aOR†	aOR††
	(CI)	(CI)	(CI)	(CI)	(CI)	(CI)	(CI)	(CI)
***Employment status (Ref*. *Standard worker)***								
**Non-standard**	0.37***	0.40***	0.68***	0.74***	0.76***	0.81***	0.89	0.93
	(0.33–0.42)	(0.35–0.46)	(0.60–0.78)	(0.64–0.84)	(0.67–0.87)	(0.71–0.93)	(0.77–1.03)	(0.80–1.09)
**Self-employed**	0.21***	0.25***	0.64***	0.71***	0.66***	0.72***	0.78***	0.81***
	(0.19–0.24)	(0.23–0.28)	(0.57–0.72)	(0.63–0.80)	(0.60–0.73)	(0.65–0.81)	(0.69–0.88)	(0.71–0.93)
**Unpaid family worker**	0.27***	0.32***	0.62***	0.69***	0.80**	0.91	0.92	0.99
	(0.22–0.32)	(0.27–0.38)	(0.51–0.74)	(0.57–0.83)	(0.67–0.96)	(0.76–1.09)	(0.76–1.12)	(0.81–1.21)
***Socio-economic factors***								
**Female **	0.86***	0.92*	1.34***	1.44***	1.05	1.08	0.78***	0.78***
	(0.79–0.94)	(0.84–1.00)	(1.22–1.47)	(1.31–1.58)	(0.96–1.14)	(0.98–1.18)	(0.70–0.87)	(0.70–0.87)
**Age**	1.06***	1.06***	1.03***	1.02***	1.05***	1.05***	1.07***	1.07***
	(1.06–1.07)	(1.05–1.07)	(1.02–1.03)	(1.02–1.03)	(1.05–1.06)	(1.04–1.05)	(1.06–1.09)	(1.06–1.08)
***Income (Ref*. *to the poorest)***								
**Poor**	1.19***	1.20***	0.93	0.94	1.08	1.07	0.94	0.97
	(1.06–1.34)	(1.06–1.36)	(0.81–1.07)	(0.82–1.08)	(0.95–1.22)	(0.94–1.21)	(0.80–1.10)	(0.82–1.14)
**Richer**	1.46***	1.44***	0.93	0.92	1.34***	1.30***	1.22**	1.22**
	(1.29–1.66)	(1.27–1.64)	(0.81–1.06)	(0.80–1.06)	(1.18–1.51)	(1.15–1.48)	(1.04–1.44)	(1.04–1.43)
**Richest**	1.70***	1.65***	1	0.98	1.61***	1.54***	1.62***	1.61***
	(1.50–1.93)	(1.45–1.88)	(0.87–1.16)	(0.85–1.14)	(1.42–1.82)	(1.36–1.75)	(1.38–1.90)	(1.37–1.88)
***Marital Status (Ref*. *to Married and stayed with a spouse)***								
**Others**	0.9	0.95	0.83**	0.81**	0.9	0.93	1.04	1.08
	(0.75–1.07)	(0.79–1.14)	(0.70–0.98)	(0.68–0.96)	(0.77–1.06)	(0.79–1.10)	(0.85–1.28)	(0.87–1.33)
**Never married**	0.75***	0.72***	0.78***	0.75***	0.55***	0.54***	0.50***	0.47***
	(0.65–0.86)	(0.62–0.83)	(0.66–0.93)	(0.62–0.90)	(0.45–0.66)	(0.45–0.66)	(0.37–0.67)	(0.35–0.64)
**Suburban**	1.22***	1.19***	1.29***	1.29***	1.12**	1.12*	1	1.02
	(1.08–1.38)	(1.05–1.35)	(1.12–1.48)	(1.13–1.49)	(1.00–1.25)	(1.00–1.25)	(0.88–1.14)	(0.89–1.16)
***Education (Ref*. *to primary school) ***								
**Secondary school **	0.99	0.96	0.72***	0.70***	1.05	1	1.07	0.99
	(0.86–1.14)	(0.83–1.11)	(0.63–0.82)	(0.61–0.81)	(0.92–1.19)	(0.87–1.14)	(0.93–1.24)	(0.85–1.16)
**Higher education**	1.47***	1.37***	0.82**	0.82**	1.43***	1.27***	1.41***	1.19*
	(1.25–1.74)	(1.14–1.64)	(0.70–0.97)	(0.68–0.97)	(1.21–1.67)	(1.06–1.51)	(1.19–1.68)	(0.98–1.45)
**Medical Aid recipients**	0.75	0.74	1.35*	1.38*	1.12	1.12	1.06	1.02
	(0.53–1.07)	(0.52–1.07)	(0.95–1.91)	(0.97–1.96)	(0.75–1.67)	(0.75–1.68)	(0.67–1.67)	(0.64–1.63)
**Private health insurance**	1.73***	1.65***	1.21***	1.19**	1.74***	1.75***	1.57***	1.59***
	(1.52–1.96)	(1.45–1.88)	(1.06–1.38)	(1.04–1.36)	(1.53–1.97)	(1.54–1.99)	(1.35–1.82)	(1.36–1.85)
***Health-related factors***								
**Present chronic disease**		1.26***		1.30***		1.31***		1.28***
		(1.14–1.41)		(1.18–1.44)		(1.18–1.45)		(1.14–1.44)
**Depressive symptom over 2 weeks**		0.83***		0.94		0.85**		0.85**
		(0.73–0.95)		(0.82–1.09)		(0.74–0.97)		(0.72–1.00)
***Work-related factors***								
**Manual work**		0.79***		0.91		0.80***		0.79***
		(0.71–0.88)		(0.81–1.03)		(0.72–0.90)		(0.68–0.92)
**Duty hours (Ref. to Day)**								
**Evening or night**		0.61***		0.88		0.79***		1.03
		(0.52–0.72)		(0.74–1.05)		(0.67–0.93)		(0.84–1.26)
**Shift or split work**		2.26***		1.89***		1.31***		1.19
		(1.83–2.80)		(1.59–2.25)		(1.07–1.60)		(0.95–1.50)
***Year effect and eligibility of the NHIS cancer screening***								
**Year (Ref. to 2007)**								
**2008**	1	1.03	1.08	1.13	1	0.96	1.09	1.07
	(0.84–1.19)	(0.84–1.25)	(0.90–1.31)	(0.93–1.39)	(0.82–1.23)	(0.78–1.19)	(0.84–1.41)	(0.82–1.42)
**2009**	0.97	1.02	0.9	0.95	1.08	1.07	1.33**	1.32**
	(0.82–1.14)	(0.85–1.22)	(0.76–1.08)	(0.79–1.15)	(0.90–1.31)	(0.88–1.30)	(1.05–1.68)	(1.03–1.69)
**2010**	1.01	1.09	0.97	1.04	1.14	1.15	1.51***	1.53***
	(0.84–1.21)	(0.89–1.32)	(0.81–1.16)	(0.86–1.26)	(0.94–1.39)	(0.94–1.41)	(1.19–1.93)	(1.19–1.97)
**2011 **	0.92	1.01	1.19*	1.28**	1.19*	1.20*	1.63***	1.64***
	(0.76–1.10)	(0.83–1.23)	(0.98–1.45)	(1.04–1.58)	(0.97–1.45)	(0.97–1.48)	(1.28–2.08)	(1.28–2.11)
**2012**	1.01	1.08	1.26**	1.36***	1.22*	1.24*	1.65***	1.69***
	(0.83–1.22)	(0.88–1.33)	(1.05–1.53)	(1.11–1.66)	(0.99–1.50)	(0.99–1.54)	(1.29–2.12)	(1.31–2.19)
**Eligibility >40**					2.95***	3.14***		
					(2.54–3.44)	(2.69–3.67)		
**Eligibility > 50**							1.85***	1.84***
							(1.53–2.23)	(1.52–2.23)
**Total**	16,509	15,816	16,509	15,816	16,471	15,782	16,437	15,748

*** p<0.01

** p<0.05

* p<0.1

† Adjusted for age, sex, income, marital status, living area, education, medical aid recipients, private health insurance, year, and eligibility for each screening

†† Adjusted for age, sex, income, marital status, living area, education, medical aid recipients, private health insurance, year, eligibility for each screening, chronic disease, depressive symptom, manual work, and duty hours.

Socioeconomic factors had effects on service utilisation. Female workers were more likely to get vaccinated, but less likely to get the regular health screening and colon cancer screening. Income-related gaps existed in the regular health screening and cancer screenings, but not for the influenza vaccination. Marital status, particularly in the ‘never-married group’, was associated with the lower uptake in all four services. Living in the suburban area showed heterogeneous association on the service utilisation. Education showed the various effect of the type of services. People with higher education year were less likely to get vaccinated, but more likely to get the other services. Interestingly, Medical aid recipients did not show higher uptake except for the vaccination, compared to people with the National health insurance services. Having private indemnity health insurance was associated with higher uptakes in all four services.

With health-related factors, having at least one chronic disease was associated with a higher uptake in all four services. Interestingly, having a depressive symptom that last for more than two weeks was associated with lower uptake of regular health screenings (aOR = 0.83, 95%CI, 0.73–0.95), stomach cancer screenings (aOR = 0.85, 95%CI, 0.74–0.97), and colon cancers (aOR = 0.85, 95%CI, 0.72–1.00).

Concerning work-related factors, manual workers were less likely to get the regular health checks and cancer screenings, but not in vaccinations. Workers who have fixed duty hours in the evening or night had the lower uptake of regular health screening and cancer screening. Interestingly, people pulling shift or having split work were more likely to get vaccinated (aOR = 1.89; 95%CI, 1.59–2.25), regular health check (aOR = 2.26; 95%CI, 1.83–2.80), and stomach cancer screening (aOR = 1.31; 95%CI 1.07–1.60).

Lastly, there was a yearly effect in the service utilisation. Compared to the baseline year, which was2007, the vaccine uptake rate improved in the year 2011 and 2012. An earlier and more substantial year effect was observed in colon cancer screening from 2009 and onwards. When the eligibility criteria on the age were controlled, it also had an influence on the service utilisation. The strongest impact was observed in the stomach cancer screenings (aOR = 3.14; 95%CI 2.69–3.67) compared to the results in the colon cancer screenings (aOR = 1.84; 95%CI 1.52–2.23).

Table 5 presents the results of cervical and breast cancer screenings. verall, NSWs and the SE showed less uptake of both services in fully-adjusted models. However, these gaps were not observable between SWs and UFWs. The probabilities of getting cervical and breast cancer screening were 17% to 16% less in NSWs, respectively, compared to those of SW. Similarly, the probabilities of getting cervical and breast cancer screening were 17% to 22% less in the SWs, respectively, compared to those of SWs.

**Table 5:** Logistic regression analyses of preventive health services for female workers.

Types of preventive health service	Cervical cancer screening within 2 years		Breast cancer screening within 2 years	
Variables	aOR†	aOR††	aOR†	aOR††
	(CI)	(CI)	(CI)	(CI)
***Employment status (Ref*. *Standard worker)***				
**Non-standard**	0.80***	0.83**	0.79***	0.84**
	(0.69–0.93)	(0.71–0.97)	(0.67–0.93)	(0.70–0.99)
**Self-employed**	0.78***	0.83**	0.72***	0.78***
	(0.67–0.92)	(0.70–0.98)	(0.60–0.86)	(0.65–0.94)
**Unpaid family worker**	0.91	0.97	0.77**	0.82*
	(0.75–1.11)	(0.79–1.19)	(0.63–0.95)	(0.66–1.02)
***Socio-economic factors***				
**Age**	1.02***	1.02***	1.04***	1.03***
	(1.01–1.03)	(1.01–1.03)	(1.03–1.05)	(1.02–1.05)
***Income (Ref*. *Poorest)***				
**Poor**	1.07	1.08	1	0.99
	(0.91–1.26)	(0.91–1.28)	(0.84–1.20)	(0.83–1.18)
**Richer**	1.29***	1.32***	1.12	1.1
	(1.09–1.53)	(1.11–1.57)	(0.93–1.34)	(0.92–1.33)
**Richest**	1.44***	1.45***	1.43***	1.36***
	(1.22–1.71)	(1.22–1.73)	(1.18–1.73)	(1.12–1.65)
***Marital Status (Ref*. *Married and stayed with a spouse)***				
**Others**	1.12	1.15	1.06	1.06
	(0.93–1.35)	(0.95–1.39)	(0.87–1.28)	(0.88–1.29)
**Never married**	0.18***	0.18***	0.32***	0.34***
	(0.14–0.24)	(0.14–0.23)	(0.24–0.44)	(0.25–0.46)
**Suburban**	0.85**	0.83***	1.16*	1.15
	(0.74–0.97)	(0.72–0.95)	(0.98–1.37)	(0.96–1.36)
***Education (Ref*. *Primary school)***				
**Secondary school **	1.26**	1.23**	1.17	1.14
	(1.05–1.51)	(1.02–1.49)	(0.97–1.41)	(0.93–1.38)
**Higher education**	1.43***	1.37**	1.57***	1.37**
	(1.12–1.82)	(1.04–1.79)	(1.23–2.00)	(1.04–1.80)
**Medical Aid recipients**	1.04	1.08	1.03	1.02
	(0.68–1.61)	(0.70–1.68)	(0.68–1.55)	(0.67–1.54)
**Private health insurance**	1.85***	1.85***	1.95***	2.01***
	(1.56–2.20)	(1.55–2.21)	(1.62–2.36)	(1.66–2.43)
***Health-related factors***				
**Present chronic disease**		1.05		1.11
		(0.90–1.23)		(0.94–1.31)
**Depressive symptom over 2 weeks**		0.95		0.88
		(0.81–1.13)		(0.74–1.05)
***Work-related factors***				
**Manual work**		0.93		0.82**
		(0.79–1.09)		(0.68–0.99)
***Duty hours (Ref*. *Day)***				
**Evening or night**		0.75***		0.77**
		(0.62–0.92)		(0.63–0.95)
**Shift or split work**		1.25		1.28
		(0.91–1.74)		(0.92–1.78)
***Year effect and eligibility of the NHIS cancer screening***				
**Year (Ref. 2007)**				
**2008**	1	0.97	1.08	1.05
	(0.79–1.27)	(0.76–1.23)	(0.83–1.42)	(0.79–1.38)
**2009**	1.01	1.05	1.25*	1.26*
	(0.80–1.27)	(0.82–1.33)	(0.97–1.61)	(0.97–1.65)
**2010**	0.96	1.01	1.19	1.22
	(0.75–1.23)	(0.78–1.30)	(0.89–1.59)	(0.91–1.63)
**2011**	0.87	0.9	1.1	1.12
	(0.68–1.11)	(0.70–1.16)	(0.84–1.45)	(0.85–1.48)
**2012**	0.83	0.87	0.99	1.01
	(0.64–1.06)	(0.67–1.13)	(0.75–1.30)	(0.76–1.34)
**Eligibility over 40**			4.34***	4.70***
			(3.46–5.44)	(3.71–5.95)
**Total**	7,411	7,105	7,413	7,108

*** p<0.01

** p<0.05

* p<0.1

† Adjusted for age, sex, income, marital status, living area, education, medical aid recipients, private health insurance, year, and eligibility for each screening

†† Adjusted for age, sex, income, marital status, living area, education, medical aid recipients, private health insurance, year, eligibility for each screening, chronic disease, depressive symptom, manual work, and duty hours.

Compared to the four outcomes in the previous analysis, there was some similarity in the effects of socioeconomic factors and work-related factors on the uptake of female cancer screenings. For example, higher education, higher income, living with a spouse, and having private health insurance were associated with the higher uptake of the services. Regarding work-related factors, workers with evening or night shifts were associated with the lower uptake, but ‘pulling a shift or having a split work' did not make a difference.

Differences were observed in local factors, health-related factors, and year effects. There was a regional gap in the uptake of cervical cancer (aOR = 0.83; 95%CI, 0.72–0.95). Moreover, unlike the positive and negative impacts of health-related factors in the previous analysis, neither having chronic disease nor depressive symptoms were associated with cervical and breast cancer screening. Unlike the colon cancer screening, there has been no improvement in the uptake of these two screenings since 2007.

## Discussion

Our study suggests that, in a nationally represented population of economically active South Korean workers, NSWs were less likely to receive all services, excluding colon cancer screening, compared to standard workers. The SE were also less likely to uptake all six services whereas UFWs were less likely to receive vaccination and regular health screening compared to SWs.

Currently, regular health examination at least once a year is a requirement for employment subscribers by the labour law. If this requirement is not met, the employer is fined from 50,000 KRW up to 150,000 KRW (about 50 to 150 USD) per person [25]. However, the results suggest that the current mandate on the regular health examination is not enough because only 48.91% of male workers and 35.45% of female workers are standard workers, who are under the mandate.

Concerning the influenza vaccination and cancer screenings, the gaps between SWs and the others were smaller than the gap in regular health checks. This is owed to the NHIS vaccination program and cancer screening program in South Korea which have contributed to improving the outcomes throughout the years. And yet, the gaps in the cancer screenings between SWs and the SE still must be acknowledged. According to a previous research, the two main reasons workers were unable to visit any clinic were ‘lack of time’ (68%) and ‘no substitute worker available in the business’ (18%) [23]. Given that 82.7% of all self-employment in South Korea is based on small businesses with less than five employees, it is evident that unmet medical needs of the SE stem from such inevitable circumstances [23].

Socioeconomic gaps also must be noted. Among the four areas covered in Table 4, female workers were less likely to get regular health screenings and colon cancer screenings, but more likely to have influenza vaccinations. No utilisation gaps existed in the stomach cancer screenings. In relation to income, there were gaps in the utilisation among income groups, and so were among education groups. Higher income and higher education had an association with higher uptake of preventive health care services.

On the other hand, workers with chronic diseases were more likely to get vaccinated, and this finding is consistent with previous studies [29, 36]. This may be due to their need for regular clinic visits and physicians’ recommendations [36]. This is in line with the South Korean government’s policy to exempt chronic disease patients from being charged for vaccinations [37]. In regard to female cancer screenings, chronic disease comorbidity was negatively associated with being screened for cervical cancer. This result is consistent with previous studies that have suggested that chronic disease is a barrier to breast and cervical cancer screening. [38, 39].

### Limitations

The current study has several limitations. First of all, due to the cross-sectional nature of this study, findings are not causal estimation [40]. We have considered addressing endogeneity by using instrument variables; however, we could not find any proper one. Secondly, the health gaps among the four groups of workers may have been exaggerated since the survey period coincides with the great recession of the world economy that resulted in a steep increase in unemployment rates. In order to overcome these problems, longitudinal data is necessary so that we could control for the individual effect as well as the change in the employment.

### Policy implication and conclusions

Our study elucidates gaps existing in the utilisation of the preventive services based on their employment status in South Korea. From the results of our study, improving access to preventive health services for NSWs, the SE, and UFWs seems to be an essential step to take. A study showed that the primary physician’s recommendation is one of the most powerful influences that enhances the uptake of cancer screenings [41]. In addition to the physician recommendation, a carefully designed health system must be implemented in order to raise the national screening rate decisively [42]. According to a meta-analysis study, the most potent intervention to increase the use of adult immunisation and cancer screening services involved organisational changes such as designating separate clinics devoted to prevention, using a planned care visit for prevention, and hiring non-physician staffs for specific prevention activities [43]. Patient financial incentives such as monthly premium reduction, as well as patient reminders by healthcare-related vouchers, text messages, and phone calls, are also presumed to be effective.

In conclusion, there are gaps in the utilisation of preventive services among South Korean workers depending on their types of employment. Therefore, improving access to preventive health care services for NSWs, the SE, and UFWs should be prioritised.
